# Cognitive Behavioral Therapy Improves the Quality of Life for Patients with Mild to Moderate Depression due to Glaucoma or Cataracts: A Retrospective Study

**DOI:** 10.62641/aep.v53i2.1673

**Published:** 2025-03-05

**Authors:** Yinchen Lin, Hong Zhang, Chunliu Shi, Na Yang, Xiaoqing Li, Jinhua Gan

**Affiliations:** ^1^School of Nursing, Southwest Medical University, 646000 Luzhou, Sichuan, China; ^2^Department of Ophthalmology, Deyang People’s Hospital, 618000 Deyang, Sichuan, China; ^3^Department of Ophthalmology, The Second People’s Hospital of Deyang, 618000 Deyang, Sichuan, China; ^4^Department of Ophthalmology, The Affiliated Hospital of Southwest Medical University, 646000 Luzhou, Sichuan, China

**Keywords:** cognitive behavioral therapy, quality of life, mild to moderate depression, glaucoma, cataracts

## Abstract

**Background:**

Individuals affected with glaucoma and cataracts are more likely to experience depressive symptoms, which can affect their overall quality of life (QOL) and functioning. Therefore, this retrospective cohort study aimed to evaluate the impact of cognitive behavioral therapy (CBT) on glaucoma or cataracts patients with mild to moderate depression.

**Methods:**

This study included patients with mild to moderate depression resulting from glaucoma or cataracts in our hospital from January 2023 to December 2023. The study participants were divided into an untreated group and a cognitive behavioral therapy group based on different intervention methods. We assessed depressive symptoms using the Center for Epidemiologic Studies Depression Scale (CES-D) and Patient Health Questionnaire-9 (PHQ-9). QOL was evaluated using the Chinese translation version of the European Quality of Life-5 Dimensions scale (EQ-5D), Short Form Health Survey (SF-36), National Eye Institute Visual Function Questionnaire-25 (NEI-VFQ-25), and Hospital Anxiety and Depression Scale-Anxiety (HADS-A). Additionally, cognitive behavioral therapy (CBT) was administered to a subgroup of patients with mild to moderate depression, and the impact of CBT on the well-being of the patients was analyzed.

**Results:**

This study included 3010 individuals, consisting of untreated patients (n = 2151) and those who received cognitive behavioral therapy (n = 859). Post-intervention analysis revealed that compared to baseline, the CES-D scores (*p* < 0.001), PHQ-9 scores (*p* < 0.001), and HADS anxiety subscale scores (*p* < 0.001) were significantly reduced in the CBT group. Furthermore, the CBT group demonstrated a significant increase in EQ-5D scores (*p* < 0.001) and SF-36 mental component scores (*p* < 0.001) post-intervention compared to baseline.

**Conclusion:**

These findings offer significant insights into the prospective effectiveness of CBT in improving depressive symptoms and QOL in individuals with glaucoma or cataracts.

## Introduction

Glaucoma and cataracts are among the leading causes of vision impairment and blindness globally, posing substantial challenges for individuals in their visual functioning and daily activities [1, 2, 3]. The impact of these conditions extends beyond physical manifestations, with increasing evidence highlighting the significant association between ophthalmic diseases and mental health issues, particularly depression [4, 5]. Studies have reported that individuals with glaucoma and cataracts are more likely to experience depressive symptoms, which can affect their overall well-being and functioning [6, 7, 8]. Therefore, it is essential to address depression not only to support mental health but also to improve visual outcomes and maintain a good quality of life (QOL).

Cognitive behavioral therapy (CBT) is widely recognized for its efficacy in addressing depressive symptoms and improving QOL across various populations [9, 10]. Individuals with chronic health conditions, such as glaucoma and cataracts, often experience increased depression and reduced overall well-being, emphasizing the need for effective interventions to address these psychological comorbidities [8, 11]. In the context of ophthalmic conditions, the impact of depression extends beyond mental health, influencing visual functioning and QOL. Despite this, the potential therapeutic role of CBT in this specific population remains unexplored. Therefore, the objective of this retrospective cohort study is to evaluate the impact of CBT on individuals with mild to moderate depression due to glaucoma or cataracts, focusing on depressive symptoms and QOL outcomes.

## Materials and Methods

### Study Design

This retrospective cohort study included patients with mild to moderate depression caused by glaucoma or cataracts from January 2023 to December 2023. The patient selection process involved multiple stages to ascertain that their preferences were duly acknowledged and considered. Initially, physicians provided exhaustive information about the available treatment options to each patient in an accessible and clear manner. They comprehensively explained the advantages and possible risks, ensuring that patients fully understood their choices. Subsequently, decisions were made jointly by patients and physicians via candid and inclusive dialogues, where they deliberated over the feasible therapies, factoring in the patient’s health status, individual values, and inclinations. Importantly, all decisions adhered to the tenets of medical ethics, ensuring the patient’s right to self-determination and informed consent throughout the decision-making process. All procedures were conducted in accordance with the Declaration of Helsinki. This study was approved by the Institutional Review Board and the Ethics Committee of Deyang people’s Hospital (approval No. 2024-04-007-H01). This retrospective study waived informed consent as it utilized de-identified patient data, ensuring no potential harm or impact on patient care. The Institutional Review Board approved this exemption, following the regulatory and ethical guidelines associated with retrospective studies.

### Eligibility Criteria of the Study Participants

Inclusion criteria for study participants were set as follows: ① Patients diagnosed with primary glaucoma/cataract [12, 13], ② those undergoing surgical treatment, ③ patients with disease duration of ≥6 months, ④ patients aged >18 years, ⑤ individuals having normal cognitive functions, capable of understanding and participating in questionnaire surveys, ⑥ those underwent the Center for Epidemiologic Studies Depression Scale (CES-D) or Patient Health Questionnaire-9 (PHQ-9) assessment during hospitalization, identifying mild to moderate depression, and ⑦ those participated in the entire management process with comprehensive medical records.

However, the exclusion criteria included: ① Presence of other severe chronic conditions such as liver or kidney disease, ② previously diagnosed depression patients who received related interventions at the time of visit or before admission, or concurrent other mental disorders, ③ presence of malignant tumors, ④ use of depression-related medications, ⑤ presence of acute eye diseases that could affect the reliability of the study (e.g., severe blepharitis, conjunctivitis, keratitis, or uveitis), ⑥ concurrent severe physical illnesses and ⑦ history of drug or alcohol dependency.

### Treatment Approach

The patients with eight weeks of cognitive behavioral therapy (CBT) included the following stages: ① Identifying patient cognitions: The patients were encouraged to express their emotions, behaviors, and thought processes to healthcare personnel through self-expression and communication, helping them identify the cognitive patterns that lead to adverse emotions. ② Shifting patient thought patterns: After identifying the patient’s cognition, any erroneous cognitive patterns were assessed based on their conditions and correct thinking patterns were guided in their daily activities. ③ Applying correct thinking patterns: Considering their real-life situations, healthcare personnel instructed patients to apply the transformed thinking patterns in their daily lives and establish rational cognitive thinking patterns.

### Data Collection

#### General Information

The patient’s general information was obtained through systematic case retrieval, such as age, duration of depression, presence of diabetes, duration of hypertension, annual family income, visual acuity, type of disease, and the number of cases with bilateral low vision (corrected visual acuity 0.05~0.3).

#### Depression Rating

All patients were assessed for depressive symptoms and severity using the PHQ-9 before treatment, after receiving treatment, and again after 8 weeks of treatment. The questionnaire uses a 0 to 3, 4-level scoring system, with a total score ranging from 0 to 27. Scores from 0 to 4 suggest no depressive symptoms, 5 to 9 signify mild depressive symptoms, 10 to 14 indicate moderate depressive symptoms, and 15 to 27 indicate severe depressive symptoms. The Cronbach’s α score was 0.78 [14].

CES-D was utilized to assess the frequency of current depressive symptoms. This scale comprises 20 items, each scored on a 0 to 3, 4-level system, evaluating the frequency of symptoms experienced in the past week. A total CES-D score of ≤15 indicates the absence of depressive symptoms, 16–19 suggests potential depressive symptoms, and ≥20 indicates definite depressive symptoms. The CES-D demonstrated high accuracy, with a Cronbach’s α value of 0.87 [15].

#### Quality of Life (QOL) Score

QOL was evaluated using the Chinese translated version of the European Quality of Life-5 Dimensions scale (EQ-5D), encompassing five dimensions of the health description system: mobility, self-care, usual activities, pain/discomfort, anxiety/depression, and the EQ-VAS visual analogue scale. This scale demonstrated a high level of accuracy, with a Cronbach’s α of 0.75 [16]. Currently, there are no corresponding utility value conversion tables for the EQ-5D index scores in China. This study used the Japanese Time Trade-Off (TTO) scoring conversion table, resulting in EQ-5D index scores ranging from –0.11 to 1.

The Short Form Health Survey (SF-36) questionnaire includes eight dimensions: physical functioning (PF), role physical (RP), bodily pain (BP), general health (GH), vitality (VT), social functioning (SF), role emotional (RE), and mental health (MH). Each dimension is scored on a scale of up to 100, with higher scores indicating superior quality of life in that domain. The SF-36 demonstrated high accuracy, with a Cronbach’s α value of 0.87 [17].

The National Eye Institute Visual Function Questionnaire-25 (NEI-VFQ-25) was utilized to evaluate vision-related QOL in patients following surgery. This questionnaire involves a 5-level scoring system, where 0 signifies “unable to perform” and 4 indicates “able to perform”, yielding a cumulative score between 0 and 100. A greater aggregate score was indicative of better postoperative vision-associated quality of life. The NEI-VFQ-25 demonstrated high accuracy, with a Cronbach’s α value of 0.89 [18].

#### Anxiety Score

The Hospital Anxiety and Depression Scale-Anxiety (HADS-A) was primarily used for screening anxiety symptoms in patients at general hospitals. This scale includes seven items, each assessed using a quartile grading system (ranging from 0 to 3), reflecting the patient’s experiences over the preceding month. Scores are interpreted as follows: a range of 0–7 indicates no symptoms, 8–10 suggests potential symptom manifestation and 11–21 confirms the presence of symptoms. A HADS-A score of ≥8 is the benchmark for evaluating anxiety-related symptoms. This scale demonstrated high accuracy, with a Cronbach’s α value of 0.753 [19].

### Data Cleaning and Management

Before data analysis, this study implemented a standardized data-cleaning protocol to identify and correct any inconsistencies, inaccuracies, or missing values. This procedure involved thoroughly examining the dataset, removing duplicating records, correcting data entry errors, and appropriately handling missing data entries. Data handling was executed utilizing the datawig and panda libraries in Python 3.6.0, employing deep neural networks to estimate missing values. Missing data were kept below 5% to minimize potential selection bias, with sensitivity analyses performed accordingly. For cases lost to follow-up, outcomes were calculated under worst-case and best-case scenarios. In the absence of substantial discrepancies, it was concluded that lost to follow-up had a negligible effect on the outcomes, enhancing the robustness of the conclusions. The final results were reported subsequently after imputing the missing values.

### The Post-Hoc Analysis

To conduct a post hoc analysis within G*Power 3.1.9.7 (University of Dusseldorf; Dusseldorf, Germany), the “Means: Difference between two independent means (two groups)” function based on *t*-tests was selected. The settings were adjusted as follows: a two-tailed test, an effect size (d) of 0.6, and an α error probability of 0.05. Following this, the respective sample sizes for both groups were then entered, and the power (1-β error probability) was computed, resulting in a power of 1.0.

### Statistical Analysis

The data were analyzed using SPSS 29.0 statistical software (SPSS Inc., Chicago, IL, USA). Categorical data were presented as [n (%)]. Continuous variables were initially assessed for normal distribution using the Shapiro-Wilk method. Normally distributed continuous data were expressed as ($\bar{x}$ ± s). A *p*-value < 0.05 was considered as statistical significance.

## Results

### Demographic Data and Basic Characteristics of the Study Participants

The study included 3010 participants, 2151 receiving no treatment and 859 receiving CBT. Upon analysis, no statistically significant differences were observed between these two groups regarding age, gender distribution, duration of depression, body mass index (BMI), presence of diabetes, hypertension, ethnicity, marital status, education level, employment status, annual household income, visual acuity, disease type, presence of low vision, family history of depression, and family history of anxiety disorder (*p* > 0.05, Table 1).

**Table 1.** **Comparison of baseline characteristics between the untreated and CBT groups**.

Parameters		Untreated (n = 2151)	Cognitive behavioral therapy (n = 859)	t/χ^2^	*p*-value
Age (years)		65.74 ± 5.67	65.82 ± 5.43	0.354	0.724
Gender (male/female) [n (%)]		579 (26.92)/1572 (73.08)	232 (27.01)/627 (72.99)	0.003	0.960
Duration of depression (months)		11.78 ± 3.86	11.95 ± 3.54	1.117	0.264
BMI		24.15 ± 3.15	23.95 ± 3.56	1.514	0.130
Diabetes [n (%)]		258 (11.99)	112 (13.04)	0.621	0.431
Hypertension [n (%)]		538 (25.01)	198 (23.05)	1.279	0.258
Ethnicity [Han, n (%)]		1934 (89.91)	790 (91.97)	3.017	0.082
Marital status				2.508	0.474
	Married [n (%)]	1445 (67.18)	598 (69.62)		
	Single [n (%)]	275 (12.78)	99 (11.53)		
	Divorced [n (%)]	329 (15.3)	118 (13.74)		
	Widowed [n (%)]	102 (4.74)	44 (5.12)		
Education level				1.224	0.542
	High school or less [n (%)]	1248 (58.02)	515 (59.95)		
	Bachelor’s degree [n (%)]	731 (33.98)	283 (32.95)		
	Higher [n (%)]	172 (8.00)	61 (7.10)		
Employment status				3.576	0.167
	Employed [n (%)]	972 (45.19)	411 (47.85)		
	Unemployed [n (%)]	486 (22.59)	168 (19.56)		
	Retired [n (%)]	693 (32.22)	280 (32.6)		
Annual household income (CNY)				0.995	0.608
	≤120,000 [n (%)]	1323 (61.51)	514 (59.84)		
	120,000–240,000 [n (%)]	703 (32.68)	297 (34.58)		
	≥240,000 [n (%)]	125 (5.81)	48 (5.59)		
Visual acuity (logMAR)		0.42 ± 0.14	0.41 ± 0.15	1.734	0.083
Disease type				4.644	0.200
	Advanced glaucoma in both eyes [n (%)]	731 (33.98)	287 (33.41)		
	Advanced glaucoma in single eye [n (%)]	430 (19.99)	201 (23.40)		
	Advanced cataract in both eyes [n (%)]	688 (31.99)	261 (30.38)		
	Advanced cataract in single eye [n (%)]	302 (14.04)	110 (12.81)		
Low vision in both eyes [n (%)]		323 (15.02)	137 (15.95)	0.412	0.521
Family history of depression [n (%)]		258 (11.99)	94 (10.94)	0.657	0.418
Family history of anxiety disorder [n (%)]		473 (21.99)	198 (23.05)	0.398	0.528

CBT, cognitive behavioral therapy; BMI, body mass index; logMAR, Logarithm of the Minimum Angle of Resolution. The exchange rate is 1 USD = 6.48 CNY.

### Depression Assessment

There were no significant differences in CES-D scores (*p* = 0.430) and PHQ-9 scores (*p* = 0.366) between the untreated baseline group and the untreated group after 8 weeks, indicating no changes in depression levels over 8 weeks (Table 2). Additionally, there were no significant distinctions in CES-D scores (*p* = 0.159) and PHQ-9 scores (*p* = 0.361) between the untreated and CBT baseline groups, indicating comparable baseline depression levels. However, post-intervention, the CBT group demonstrated significantly lower CES-D scores (*p* < 0.001) and PHQ-9 scores (*p* < 0.001), indicating a substantial improvement in depressive symptoms following CBT compared to baseline.

**Table 2.** **Assessment of depression between the untreated and CBT groups**.

Parameters	U^a^	U^b^	C^a^	C^b^	T^a^	*p* ^a^	T^b^	*p* ^b^	T^c^	*p* ^c^
CES-D score	21.15 ± 4.67	21.04 ± 4.12	21.41 ± 4.32	18.98 ± 8.25	0.819	0.413	1.409	0.159	9.484	<0.001
PHQ-9 score	12.74 ± 3.29	12.64 ± 4.26	12.86 ± 3.15	8.96 ± 3.52	0.862	0.389	0.915	0.361	22.442	<0.001

U^a^, untreated (baseline) (n = 2151); U^b^, untreated (after 8 weeks) (n = 2151); C^a^, cognitive behavioral therapy (baseline) (n = 859); C^b^, cognitive behavioral therapy (post-intervention) (n = 859); T^a^, *t* (U^a^ vs U^b^); *p*^a^, *p* (U^a^ vs U^b^); T^b^, *t* (U^a^ vs C^a^); *p*^b^, *p* (U^a^ vs C^a^); T^c^, *t* (U^b^ vs C^b^); *p*^c^, *p* (U^b^ vs C^b^). CES-D, Center for Epidemiologic Studies Depression Scale; PHQ-9, Patient Health Questionnaire-9.

### Quality of Life

We did not find significant differences between the untreated baseline group and the untreated group after 8 weeks (*p* > 0.05). Similarly, there were no significant differences between the untreated baseline group and the CBT baseline group (*p* > 0.05). However, the EQ-5D scores showed a significant increase post-intervention compared to baseline (*p* < 0.001), indicating enhanced overall quality of life. Additionally, the SF-36 mental component scores demonstrated a significant improvement post-intervention compared to baseline (*p* < 0.001), reflecting enhanced mental well-being following CBT. Furthermore, the HADS anxiety scores showed a significant improvement post-intervention compared to baseline (*p* < 0.001). However, there were no statistically significant differences in the SF-36 physical component scores and NEI-VFQ-25 composite scores when comparing the untreated group to the CBT group at baseline and post-intervention (*p* > 0.05, Table 3).

**Table 3.** **Quality of life assessment between the untreated and CBT groups**.

Parameters	U^a^	U^b^	C^a^	C^b^	T^a^	*p* ^a^	T^b^	*p* ^b^	T^c^	*p* ^c^
EQ-5D Score	0.71 ± 0.23	0.72 ± 0.19	0.72 ± 0.26	0.75 ± 0.26	1.555	0.120	1.037	0.300	3.501	<0.001
SF-36 physical component	46.18 ± 3.45	46.24 ± 3.98	45.92 ± 3.67	46.15 ± 3.46	0.528	0.597	1.833	0.067	0.581	0.561
SF-36 mental component	41.85 ± 5.98	42.07 ± 5.07	42.11 ± 5.45	43.11 ± 9.06	1.301	0.193	1.104	0.270	3.986	<0.001
NEI-VFQ-25 composite score	70.97 ± 8.68	70.72 ± 7.93	71.22 ± 8.55	71.36 ± 10.12	0.986	0.324	0.717	0.474	1.841	0.066
HADS anxiety score	11.04 ± 3.15	11.15 ± 2.81	10.84 ± 2.56	10.58 ± 2.43	1.209	0.227	1.655	0.098	5.217	<0.001

U^a^, untreated (baseline) (n = 2151); U^b^, untreated (after 8 weeks) (n = 2151); C^a^, cognitive behavioral therapy (baseline) (n = 859); C^b^, cognitive behavioral therapy (Post-Intervention) (n = 859); T^a^, *t* (U^a^ vs U^b^); *p*^a^, *p* (U^a^ vs U^b^); T^b^, *t* (U^a^ vs C^a^); *p*^b^, *p* (U^a^ vs C^a^); T^c^, *t* (U^b^ vs C^b^); *p*^c^, *p* (U^b^ vs C^b^). EQ-5D, European Quality of Life-5 Dimensions scale; SF-36, Short Form Health Survey; NEI-VFQ-25, National Eye Institute Visual Function Questionnaire-25; HADS, Hospital Anxiety and Depression Scale.

## Discussion

This retrospective cohort study aimed to evaluate the impact of CBT on individuals with mild to moderate depression resulting from glaucoma or cataracts. Depressive symptoms are prevalent in individuals with chronic health conditions such as glaucoma and cataracts, often alleviating quality of life and mental well-being [20, 21, 22]. Our findings align with previous research demonstrating the efficacy of CBT in improving depressive symptoms across various populations [23]. The significant reduction in CES-D and PHQ-9 scores post-intervention in the CBT group compared to baseline suggests the potential clinical utility of CBT in managing depression associated with ophthalmic conditions.

In addition to enhancements in depressive symptoms, the post-intervention analysis revealed a significant increase in EQ-5D scores, indicating an overall improvement in QOL following CBT. Individuals with ophthalmic conditions may face reduced participation in activities they previously enjoyed due to vision-related limitations, which can lead to a decrease in overall QOL and an increased risk of developing depressive symptoms [24, 25]. CBT includes behavioral activation techniques that encourage individuals to re-engage in meaningful activities and social interactions, counteracting the potential social isolation and withdrawal commonly associated with vision loss [26, 27]. Furthermore, the SF-36 mental component scores demonstrated a statistically significant improvement post-intervention, indicating enhanced mental well-being in the CBT group. These findings are consistent with existing literature highlighting the positive effects of CBT on QOL and mental health outcomes in individuals with various medical conditions [28, 29]. CBT aims to identify and modify negative thought patterns and cognitive distortions [30, 31, 32]. Individuals with glaucoma or cataracts may experience a sense of loss, fear of progressive vision impairment, and anxiety about the impact of their condition on daily functioning [33, 34]. CBT helps individuals challenge and reframe these negative thoughts, developing adaptive strategies to manage the psychological impact of their condition.

These findings indicate that the efficacy of CBT in improving depressive symptoms and QOL in individuals with mild to moderate depression due to glaucoma or cataracts may be due to its ability to alleviate anxiety and improve overall well-being. The structured nature of CBT, which focuses on identifying and modifying negative thought patterns and cognitive distortions, may contribute to the observed improvements [35]. By promoting adaptive coping strategies and challenging negative beliefs related to vision impairment, CBT may mitigate the psychological impact of ophthalmic conditions, thereby leading to reduced depressive symptoms and enhanced quality of life [26]. CBT aims to break the negative cycle by deconstructing the problems that cause anxiety or fear, helping patients alter negative patterns, enhance mood, and manage worries more effectively. Furthermore, the behavioral activation techniques employed in CBT may facilitate individuals to re-engage in meaningful activities and social interactions, counteracting the social isolation and withdrawal often associated with vision loss [36]. These theoretical observations align with previous research indicating the beneficial effects of CBT on mental health outcomes in various populations [37]. Future investigations utilizing neuroimaging techniques or qualitative assessments could provide further insights into the neurobiological and psychosocial mechanisms through which CBT shows therapeutic effects on individuals with glaucoma or cataracts.

It is important to note that the present study possesses several strengths, including a robust sample size and a comprehensive assessment of depressive symptoms and quality of life using validated measures. Furthermore, the retrospective design enabled the evaluation of real-world clinical outcomes, thereby enhancing the external validity of the findings.

However, there was no correlation between CBT and the SF-36 physical component scores, and NEI-VFQ-25 composite scores. CBT primarily relies on the patient’s willpower and focuses on psychological support, so it has minimal effect on physical health. The recovery of visual function needs rehabilitation training and drug treatment. Therefore, the impact of CBT on physical health was not evident.

However, it is crucial to recognize a few limitations of the study. Firstly, the retrospective design limits the ability to draw causal inferences, and the influence of confounding variables cannot be ruled out. Additionally, the absence of a control group and randomization hinders the ability to establish a direct causal relationship between CBT and the observed improvements. Future prospective studies with randomized controlled designs are required to provide further insight into the efficacy of CBT in this population. Another important consideration is the generalizability of the findings. The study cohort primarily consisted of individuals with mild to moderate depression associated with glaucoma or cataracts, which may limit the applicability of the findings to those with severe depression or other ophthalmic conditions. Furthermore, the study was conducted at a single institution, and the demographic characteristics of the sample may not reflect the broader population. Future research should aim to replicate these findings across diverse clinical settings and patient populations to enhance the generalizability of the results.

## Conclusion

In conclusion, this retrospective cohort study provides evidence for the potential efficacy of CBT in improving depressive symptoms and QOL in individuals experiencing mild to moderate depression due to glaucoma or cataracts. The findings highlight the significance of addressing mental health in the context of ophthalmic conditions and suggest the role of CBT as a potential therapeutic modality for this population. Further research, particularly prospective controlled studies, is warranted to confirm these findings and support evidence-based interventions for depression in individuals with ophthalmic conditions.

## Availability of Data and Materials

Data to support the findings of this study are available on reasonable request from the corresponding author.
